# Modeling the Population Dynamics and Management of Italian Ryegrass under Two Climatic Scenarios in Brazil

**DOI:** 10.3390/plants9030325

**Published:** 2020-03-04

**Authors:** Fortunato D. B. Pagnoncelli, Michelangelo M. Trezzi, Jose L. Gonzalez-Andujar

**Affiliations:** 1 Department of Agronomy, Federal University of Technology–Paraná, Pato Branco 85503-390, Brazil; trezzi@utfpr.edu.br; 2 Department of Crop Protection, Institute for Sustainable Agriculture (CSIC), Spain and International Laboratory on Global Change (LINCGlobal) (CSIC), 14005 Córdoba, Spain; andujar@cica.es

**Keywords:** *Lolium multiflorum*, stochastic model, climate change, seed bank, management strategies

## Abstract

Italian ryegrass (*Lolium multiflorum L.*) is an annual grass widely distributed in cultivated crops around the world. This weed causes significant yield reduction in many crops and has developed herbicide resistance. The aim of this study was to develop a cohort-based stochastic population dynamics model that integrates both emergence (thermal time) and dynamic population models as a tool to simulate the population dynamics of susceptible and resistant populations of *L. multiflorum* under the effects of climate change. The current climate scenario and the increase in the average air temperature by 2.5 °C were considered. Chemical and cultural management strategies commonly used in the South Region of Brazil during the winter and summer seasons were incorporated into the model. In the absence of control and under the current climate conditions, the seed bank population grew until reaching an equilibrium density of 19,121 ± 371 seeds m^−2^ for the susceptible and 20463 ± 363 seeds m^−2^ for the resistant populations. Considering the second climate scenario, the seed bank reaches an equilibrium density of 24,182 ± 253 seeds m^−2^ (+26% in relation to the current scenario) for the susceptible population and 24,299 ± 254 seeds m^−2^ (+18% in relation to the current scenario) for the resistant one. The results showed that the effect of the rise in temperature implies an increase in population in all the management strategies in relation to the current climate scenario. In both climate scenarios, the strategies based on herbicides application controlling cohorts 1 and 2 were the most efficient, and cropping systems including winter oat-soybeans rotation had a smaller impact on the *L. multiflorum* seed bank than crop rotations including winter wheat or summer corn. Crop rotations including wheat and corn for *L. multiflorum* management as an adaptive strategy under the future climate change are suggested.

## 1. Introduction

*Lolium multiflorum L.* is an annual species widespread in cultivated areas of the world [1]. This species has characteristics that allow it to be used for different purposes in agricultural systems, and it is widely used in intensive systems, like in the South Region of Brazil. This weed causes significant yield reduction in many crops [2] and has developed herbicide resistance. The species has stood out for its development of herbicide resistance, which has hindered management and increased production costs [2]. The first case of resistance to herbicides in Brazil was in 2004 (i.e., to glyphosate) [3]. The intensive and sometimes inappropriate use of ALS-inhibiting herbicides and ACCase in the last few years has led to the evolution of resistance to these mechanisms as well.

Population dynamics models allow future predictions of the behavior of a population. Such models are based on the study of demographic parameters, obtained from evaluations carried out in specific stages of plant development, such as seed mortality in the seed bank, emergence and survival rate of seedlings, and the production and dispersion of seeds. The models permit the simulation of management strategies and their assessment. This is an important feature mainly from an economic perspective, because of the expense of long-term field experiments and the amount of time required to obtain the results. The objective of using mathematical models is not to make an exact representation of the phenomenon, but rather to characterize the problem and extrapolate the consequences of management strategies to population dynamics [4]. The demographic parameters of a model can be attributed to fixed values [5]. However, agricultural systems are constantly undergoing abiotic and biotic disturbances caused by the environment; these disturbances may impact the model parameters. Therefore, randomness can be included through a stochastic model [6] which aims to represent the fluctuations that may occur over the years.

Several studies have already been carried out to assess the long-term population behavior of different weed species, including *Lolium rigidum* [5,6,7]. To the best of our knowledge, there are no studies of this kind on *L. multiflorum.* Moreover, there are still few studies comparing the dynamics of susceptible and herbicide-resistant populations [8]; in most cases, projections are made of the evolution of the herbicide-resistant frequency [9,10]. Several studies have shown that plants resistant to herbicides show morphophysiological changes in comparison to susceptible ones [11,12,13].

Studies assessing climate change show that from the years 1850 until 2015, the Earth’s average temperature has increased by approximately 0.8 °C [14], and the projection for the year 2050 is that it will increase by 2.0 °C [15]. The rise in temperature is linked to the elevation of atmospheric CO_2_, and it has an influence on the distribution of precipitation around the world. These estimates generally suggest that temperature increases and changes in the water regime would reduce the temporal distribution of seedling emergence, which would occur in more specific time-periods [16,17].

Weed population models have proved to be very useful for evaluating weed management strategies and developing decision-making tools [4]. However, they have hardly ever been used to study the impact of climate change on the management of weed populations. This study aimed to develop a cohort-based stochastic population model that integrates both emergence (thermal time) and population dynamics models as a tool to simulate the effect of commonly used management strategies in Brazil on the population dynamics of susceptible and resistant populations of *L. multiflorum* under the effects of climate change.

## 2. Results

### 2.1. Scenario 1 (Average Temperature 2007–2017)

**Figure 1:**
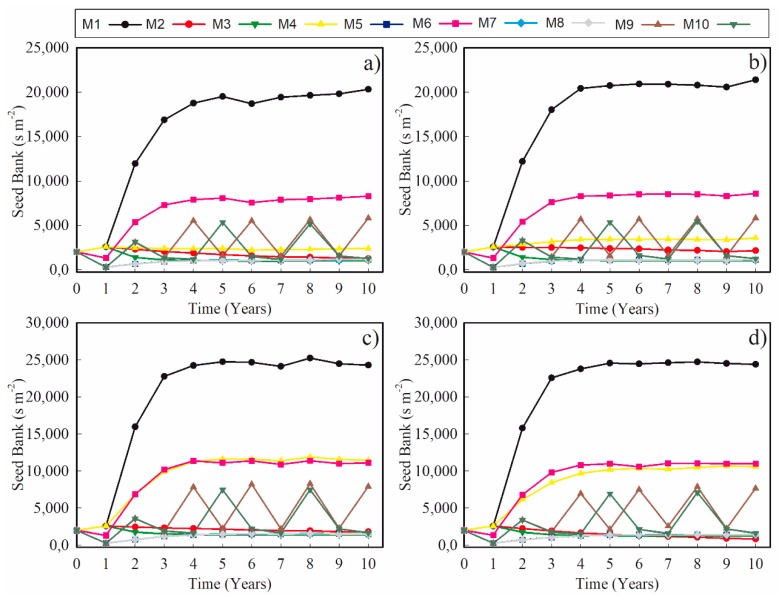
Average dynamics of *Lolium multiflorum* seed bank. For Scenario 1, mean temperature between 2007 and 2017 for populations susceptible (**a**) and resistant to glyphosate (**b**), and for Scenario 2, with an expected average daily temperature increase in 2.5 °C for populations susceptible (**c**) and resistant to glyphosate (**d**). Under different management strategies. **M1** = Null, **M2** = PosLC1 + PosEC2, **M3** = PosLC1 + PosLC2 + PosEC3, **M4** = PosEC2 + PosLC3, **M5** = Wheat/Soybean, **M6** = Oat/Soybean, **M7** = Oat/Corn, **M8** = Wheat/Soybean/Oat/Corn, **M9** = Oat/Soybean/Oat/Corn, **M10** = Wheat/Soybean/Oat/Corn/Oat/Soybean.

Strategy M1 represents the development of the life-cycle with no controls. In the absence of control practices, the seed bank grew until reaching a population equilibrium of 19,121 ± 371 seeds m^−2^ and 20,463 ± 363 seeds m^−2^ for the susceptible and resistant populations, respectively (Figure 1). These values can be considered as the carrying capacities of *L. multiflorum*.

The most common method of controlling *L. multiflorum* in Brazil is by using the post-emergence application of herbicides. Our simulations indicate that chemical managements (M2, M3, and M4) had a high impact on the seed bank and were able to reduce the carrying capacity of the system ranging between 88% (M4; 2339 ± 25 seeds m^−2^; mean ± standard deviation) and 95% (M3; 1009 ± 15 seeds m^−2^) for susceptible populations and between 83% (M4; 3409 ± 42 seeds m^−2^) and 95% (M3; 1045 ± 18 seeds m^−2^) for resistant ones (Figure 1).

The management strategies based on crop rotations were as effective as herbicides in some cases. The control ranged from 59% (M6; 7877 ± 111 seeds m^−2^) to 94% (M8; 1059 ± 31 seeds m^−2^) for susceptible populations and between 59% (M6; 8339 ± 108 seeds m^−2^) and 95% (M8; 1091 ± 15 seeds m^−2^) for resistant ones (Figure 1).

Considering all the strategies, M3 (based on post-emergence late control in C1 and C2 and post-emergence early control in C3) was the most effective management strategy for both susceptible and resistant populations and the worst one was the strategy based on an oat/soybean rotation (M6).

### 2.2. Scenario 2 (2.5 °C Increase in Average Temperature)

With the expected elevation in temperature as a consequence of global climate change, an increase in the environment carrying capacity was observed when compared to Scenario 1 (Figure 1). The increase was 26% for the susceptible population (19,121 ± 371 => 24,182 ± 253 seeds m^2^), 18% for the resistant population (20,463 ± 363 => 24,299 ± 255 seeds m^2^). Similarly, our results show that the effect of the rise in temperature implies an increase in population in all the management strategies in relation to Scenario 1 (Figure 1). However, the reduction efficiency in relation to the baseline strategy was approximately the same. The control for herbicides application ranged from 58% (M4; 10,067 ± 258) to 95% (M2; 1293± 128 seeds m^2^) for the susceptible population and from 53% (M4; 11,319 ± 213) to 94% (M3; 1425 ± 18 seeds m^2^) for resistant ones. With crop rotations, the control varied between 55% (M6; 10,763 ± 149 seeds m^2^) and 94% (M7; 1335.68 ± 41.21 seeds m^2^) for susceptible populations and between 54% (M6; 11,036 ± 140 seeds m^2^) and 94% (M8; 1462 ± 40 seeds m^2^) for resistant ones.

Strategy M3 with post-emergence late control in cohorts C1 and C2 and post-emergence early control in C3 was the most effective one for resistant populations and strategy M2 with post-emergence late control in C1 and post-emergence early control in C2 was the most effective one for susceptible ones.

## 3. Discussion

The simulations were performed using the main management strategies deployed by farmers in the South Region of Brazil and involved both winter and summer crops. The stochastic nature of the model allows the effect of the demographic parameter variability to be evaluated [6], describing the effect of biotic and abiotic factors on the system. The seed bank observed in the baseline Scenario 1 (M1) was 19,121 ± 371 seeds m^−2^ (susceptible population) and 20,463 ± 363 seeds m^−2^ (resistant population). These results are in agreement with the seed bank observed in farming areas for *L. multiflorum* [18]. These population levels are high enough to cause severe yield losses if the weeds are not controlled [19].

Strategies exclusively dependent on chemical management (M2 and M3) were highly efficient and were able to suppress the seed bank by roughly 95% with respect to the baseline of each population in both climate scenarios. However, the potential for seed bank reduction by strategy M4 (based on post-emergence late control in C1 and C3) was less than 89% of the control. The expected temperature increase directly influences the speed of soil’s thermal accumulation, which is reflected in the anticipation of the seedling emergence. The soil temperature increase promotes synchronization and reduces the temporal distribution of seedling emergence [16]. As observed, this may have implications for *L. multiflorum* management, especially for strategy M4, because the increase in temperature (Scenario 2) decreases the strategy’s efficiency, for both the susceptible and herbicide-resistant populations, with respect to the baseline. In Scenario 2, the increase in temperature results in an increase of the seedling emergence speed in relation to Scenario 1. Therefore, the *L. multiflorum* population increases in the first cohort and reduces in the third one. This circumstance suggests the need for early application of control measures and, in this way, to reduce the population of the first cohort that is the most competitive with the crop [20].

The management strategies based on crop rotations were in some cases slightly less efficient than strategies based on herbicide applications. Management strategies with wheat and corn rotation (M5, M7, and M8) have shown to be very effective in suppressing the seed bank over the long term. In fact, *L. multiflorum* is controlled with pre- and post-emergence herbicides in wheat and with post-emergence herbicide in corn. The management strategies with oats followed by soybeans (M6, M9, and M10) showed a lesser potential for suppressing the *L. multiflorum* seed bank. This can be mainly attributed to the absence of post-emergence herbicides for the control of ryegrass in oats [21].

The results show that chemical management was more effective in controlling *L. multiflorum,* although chemical control measures could become less effective due to a change in the external environment (drier and warmer conditions) or changes in the anatomy, growth physiology, and phenology of the target weed flora as a consequence of climate change [22]. On the other hand, some crop rotation strategies were just as efficient (roughly 95%) as the herbicide application, in both scenarios. Therefore, it is suggested to use crop rotations, including wheat and corn, for *L. multiflorum* management as an adaptive strategy under the future climate change.

Climate change is one of the most important problems faced by farmers, and the use of models can give an insight into the long-term dynamics of weed populations. Population dynamics approaches can play a key role by filling eco-physiology and climate niche study gaps. Our results show an increase in resistant and susceptible populations, suggesting a future worsening infestation of this species in Brazil. The simulated strategies are commonly used among Brazilian farmers, but despite the high level of control offered in some cases, they do not seem to be sufficient to achieve an efficient control of *L. multiflorum* under the climate change scenarios considered.

Although susceptible and resistant populations *of L. multiflorum* differ in the number of seeds produced per plant at low densities, the seed bank’s carrying capacity of susceptible and resistant populations was similar. In fact, with the increase in plant density, a reduction in the difference in seed production capacity per plant between populations was observed [23]. *Bassia scoparia* populations susceptible and resistant to glyphosate, although differing in emergence rates, show no differences in their seed bank after stabilization [8]. However, differences between populations were observed when considering only the use of glyphosate, by which susceptible plants would be controlled and resistant plants would survive and reproduce.

Further development of the model should include checking its accuracy under field conditions. The models can easily be extended to allow the input of parameters related to the cost of each management, as well as potential grain yield losses due to the presence of *Lolium* plants. Another possibility is the incorporation of the likelihood of herbicide resistance into different management strategies, allowing future predictions of resistance evolution in the area [24] or be included in a decision support system to develop decision-making tools [25].

## 4. Materials and Methods

**Figure 2:**
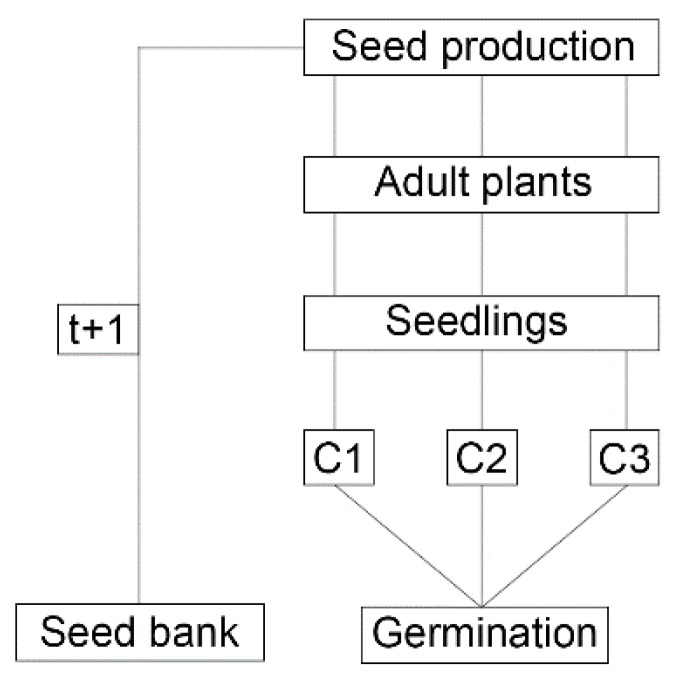
Life cycle of *Lolium multiflorum* with three cohorts (C1, C2, and C3).

The population dynamics of *L. multiflorum* was modeled according to a stochastic model that considered three seedling cohorts (March, April, and June) (Figure 2), two populations (glyphosate-resistant and susceptible) and two climate scenarios. Scenario 1: Average air temperature observed over a 10-year period (2007–2017). Scenario 2: The same as Scenario 1 with a temperature increase of 2.5 °C. The three plant cohorts were defined as representing the main seasons of plant establishment in the South Region of Brazil, at the beginning, middle, and end of the season. The pollen flow between susceptible and resistant populations was disregarded, aiming to demonstrate only the behavior of purely susceptible and purely resistant populations.

### 4.1. Plant Emergence

The number of seedlings emerging in each plant cohort was determined by the accumulated soil thermal time (TT), according to the Gompertz model (Equation (1)) (Pagnoncelli not published).

*y_d_* = 100 *exp*(−*exp*(−0.0151 (*TT* − 444.20))) (1)

where *y_d_* is the percent of seedlings emerged.

The cumulative thermal time in degrees day was calculated by:

*TT* = ∑^*n*^_*i*=1_(*Tmean* − *Tbase*) (2)

where *n* is the number of days after sowing, *Tmean* is the average daily soil temperature (°C) and *Tbase* is the lowest temperature (°C) at which the seed can germinate. The *Tbase* temperature was set at 1.9 °C [26].

The soil TT was estimated based on the average daily air temperature (AT) over a 10-year period (2007–2017) (TT = 0.84AT + 3.81; R^2^ = 0.91) for the city of Pato Branco (26°10′32”S, 52°41′11”W), Brazil. Air and soil temperature are highly correlated and in the absence of soil temperature, the air temperature can be used to predict the seedling emergence [27]. The thermal accumulation count started on March 10th, the date considered for the emergence of the first cohort. Based on the thermal accumulation observed, Scenario 1 was created.

It is expected that by the year 2050, the average daily temperature for Southern Brazil will have increased by 2.5 °C [28], which may have a direct impact on the seedling emergence flow and, consequently, on the periods of plant establishment in the field (cohorts). The information was incorporated into the model, creating Scenario 2.

The number of seedlings emerging in each cohort was determined by:

*SDL_i_* = (*y_d_*/100) *SDL* (3)

and *SDL_i_* is the total seedlings emerged (seedlings m^−2^) in each cohort (*i* = 1… 3), *y_d_* is the percent of seedling emerged until day *d*, and *SDL* are the seedlings emerged (seedlings m^−2^).

The number of seedlings with a potential emergence from the seed bank in year *t* was obtained by:

*SDL* = *SB e* (4)

where *SB* is the seed bank (seeds m^−2^) and *e* is the emergence rate.

### 4.2. Seedling Survival

Seedlings are exposed to biotic and abiotic factors that can vary in each cohort; this has a direct effect on survival and the number of adult plants. The number of surviving seedlings that reach the adult stage in each cohort is determined by:

*AP_i_* = *SDL_i_ sdls_i_* (1 − *rc*) (5)

where: *AP_i_* is the number adult plants (plants m^−2^) in each cohort, *sdls_i_* is the seedling survival in each cohort, and *rc* is the rate of control by the herbicide.

### 4.3. Seed Production

Seed production per plant is density dependent according to the hyperbolic model [29] and is subject to the cohorts and the pollination rate because the plants are obligatorily cross-pollinating. The seed production per plant was determined by:

*SP_i_* = *f*(1 − *fr_i_*) (1 − *lp*) (1 − *rc*)/(1 + *bAP_i_*) (6)

where *SP_i_* is the seed production plant^−1^ in each cohort, *f* is the max seed production for an isolated plant, *fr* is a factor reduction of fecundity (cohorts have different fecundities), *b* is the area required for a plant to produce *f* seeds, and *lp* (loss in pollination) is reduction in fecundity as a result of a pollination deficit.

The seeds produced can be lost (removed from the field) in different ways (e.g., predators). The total number of seeds produced (*TSP_i_*) in each cohort (seeds m^−2^) was determined by:

*TSP_i_* = (*SP_i_ AP_i_*) (1 − *l*) (7)

where *l* is the seed losses.

The total number of seeds produced in the 3 cohorts (*TSP*; seeds m^−2^) returning to the seed bank was obtained by:

*TSP* = ∑^*i*^_*t*=1_(*TSP_i_*) (8)

### 4.4. Seed Bank

The seed bank (*SB*; seeds m^−2^) in time *t* is given by,

*SB_t_* = *SB*_*t*−1_ (1 − *e*_*t*−1_) (1 − *sm*) + *TSP*_*t*−1_ (1 − *cr*) (9)

where *SB*_*t*−1_ is the seed bank in the previous year (seeds m^−2^), *e*_*t*−1_ is the emergence rate, *sm* is the seed bank mortality, *TSP_t−1_* is the total seed production (seeds m^−2^) in the previous year, and *cr* is the crop rotation effect when applied.

### 4.5. Model Parameters

Parameters were estimated with data obtained from experiments conducted in Pato Branco (Brazil) [30] and from the literature (Table 1). In all the simulations, an initial seed bank of 2000 seeds m^−2^ was considered [31].

Demographic and control parameters are subject to variations under field conditions, so they were considered stochastic. We modeled each parameter based on rates (except seed mortality and seed losses) by simulating its value as a random variable with a beta distribution, with a mean of µ and a standard deviation of σ and with a normal distribution for max seed by plants fecundity (*f*).

The control rates were obtained from published studies for *L. multiflorum,* and in the absence of information, data from *L. perenne* were used. The latter being the *Lolium* species with the highest morphological similarity to *L. multiflorum* [31]. Management strategies and control rates were the same in both populations. For chemical management, the application of clethodim + glyphosate was considered as they have potential to control 98% of young plants and 91% of adult ones [33].

The simulations were run for a 10-year time period with 100 repetitions each. The model was implemented in Excel^®^ (a copy is available upon request).

### 4.6. Assessing Management Strategies

Several management strategies (cultural and chemical) were simulated (Table 2).

Strategy M1 does not consider any type of management and is considered as being the baseline for comparison with other management strategies.

**Figure 3:**
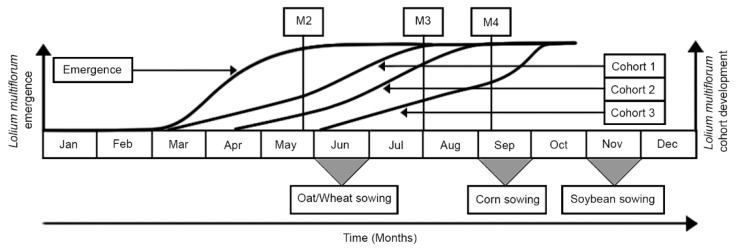
Diagram used to describe *L. multiflorum* management strategies. The emergence flow and development of each cohort are described in this work, while the crops sowing is adjusted according to Galvan et al., (2015) [31].

The management strategies M2 to M4 rely only on the early post-emergence application of herbicide (before bloom) and late applications (after bloom) in different cohorts. The schematic representation of emergence flow, establishment and development of each cohort, and the management strategies used are shown in Figure 3 (adapted from Galvan et al., 2015) [31].

The cultural strategies are based on crop rotations (M5 to M10) (Table 2). The effect of crop rotation systems was quantified through their direct impact on the seed bank. These cropping systems were chosen because they are representative of what actually occurs in the South Region of Brazil. The combination of crop rotations was chosen because farmers are unlikely to cultivate the same crop in the summer for several consecutive years; in fact, this practice is not recommended by agronomists. In this model, the alternation of different crops in the summer shows with greater accuracy what actually happens in the field and provides the reader with a better understanding of this information in the long term.

## Figures and Tables

**Table 1:** Parameters used in the model. (sd: standard deviation).

Parameter		Susceptible		Resistant		Reference
		Mean	sd	Mean	sd	
Seed Bank						
Seed Mortality	*Sm*	0.49	-	0.49	-	[31]
Emergence rate	*e*	0.73	0.04	0.73	0.04	[30]
Seedlings						
Seedling Survival	*sdls_i1_*	0.02	0.02	0.04	0.03	[30]
Seedling Survival	*sdls_i2_*	0.03	0.02	0.05	0.03	[30]
Seedling Survival	*sdls_i3_*	0.11	0.05	0.16	0.07	[30]
Seed Production						
Factor Reduction	*fr_i1_*	0.07	0.07	0.02	0.06	[30]
Factor Reduction	*fr_i2_*	0	-	0	-	[30]
Factor Reduction	*fr_i3_*	0.46	0.1	0.34	0.12	[30]
Max Seed Produced per Plant	*f*	20300	1212	13830	1305	[30]
Area to Produce *f* Seeds	*b*	0.17	0.03	0.12	0.03	[30]
Losses in Pollination	*lp*	0.88	0.05	0.88	0.05	[30]
Seed Losses (Standard Harvest)	*sl*	0.19	-	0.19	-	[5]
Management						
Control rate (Post Emergence Early)	*rc_postE_*	0.98	0.005	0.98	0.005	[32]
Control rate (Post Emergence Late)	*rc_postL_*	0.91	0.008	0.91	0.008	[32]
Crop Rotation Wheat/Soybean	*cr_1_*	0.89	0.03	0.89	0.03	[31]
Crop Rotation Oat/Soybean	*cr_2_*	0.48	0.13	0.48	0.13	[31]
Crop Rotation Oat/Corn	*cr_3_*	0.89	0.03	0.89	0.03	[31]

**Table 2:** Simulated chemical and cultural strategies for the management of *L. multiflorum*.

Management	Year 1		Year 2		Year 3	
	Winter	Summer	Winter	Summer	Winter	Summer
M1	Null					
M2	PostLC1 + PostEC2					
M3	PostLC1 + PostLC2 + PostEC3					
M4	PostLC2 + PostLC3					
M5	Wheat	Soybean				
M6	Oat	Soybean				
M7	Oat	Corn				
M8	Wheat	Soybean	Oat	Corn		
M9	Oat	Soybean	Oat	Corn		
M10	Wheat	Soybean	Oat	Corn	Oat	Soybean

PosE: post emergence early; PosL: post emergence late; C1, C2, and C3 are cohorts.
